# Design of Neutron Microscopes Equipped with Wolter Mirror Condenser and Objective Optics for High-Fidelity Imaging and Beam Transport

**DOI:** 10.3390/jimaging6100100

**Published:** 2020-09-27

**Authors:** Muhammad Abir, Daniel S. Hussey, Boris Khaykovich

**Affiliations:** 1 Nuclear Reactor Laboratory, Massachusetts Institute of Technology, Cambridge, MA 02139, USA; miabir@mit.edu; 2 Physical Measurement Laboratory, NIST, Gaithersburg, MD 20899, USA; daniel.hussey@nist.gov

**Keywords:** neutron focusing mirrors, Wolter optics, neutron imaging

## Abstract

We present and compare the designs of three types of neutron microscopes for high-resolution neutron imaging. Like optical microscopes, and unlike standard neutron imaging instruments, these microscopes have both condenser and image-forming objective optics. The optics are glancing-incidence axisymmetric mirrors and therefore suitable for polychromatic neutron beams. The mirrors are designed to provide a magnification of 10 to achieve a spatial resolution of better than 10 μm. The resolution of the microscopes is determined by the mirrors rather than by the L/Dratio as in conventional pinhole imaging, leading to possible dramatic improvements in the signal rate. We predict the increase in the signal rate by at least two orders of magnitude for very high-resolution imaging, which is always flux limited. Furthermore, in contrast to pinhole imaging, in the microscope, the samples are placed far from the detector to allow for a bulky sample environment without sacrificing spatial resolution.

## 1. Introduction

Neutron radiography is a collection of fast-developing imaging techniques that use the penetrating power of thermal and cold neutrons for elucidating the internal structure of materials and devices, from quantum magnets to running internal combustion engines [1,2]. This paper is concerned with attenuation-based and polarized imaging (as opposed to phase-based imaging, such as phase-contrast imaging or grating interferometry). The attenuation can be due to the sample or by the magnetic analyzer downstream of the sample if a polarized beam is used for magnetic imaging. Important applications of this technique include imaging of fuel cells [3], visualization of hydrogen blisters in iron [4], energy-selective (or Bragg-edge) imaging [5], visualization of magnetic fields and domains [6,7], nuclear fuel pins [8,9], porous and geological materials [10], etc. The need for both high spatial and temporal resolution is especially acute in operando studies of proton-exchange membrane fuel cells, where membranes that are a few microns thick are imaged through a thick metal casing, or when imaging hydrogen blisters. On the other hand, imaging gas bubbles during boiling requires millisecond time resolution [11,12]. The fundamental limitation of a relatively low neutron flux cannot be lifted. The flux of new state-of-the-art neutron research facilities has been improving at a rate of about a 10-fold increase every 20 years and at a huge cost [13,14]. Therefore, developing and improving neutron-beam optics is significantly important for expanding the reach of neutron methods. Neutron beams present unique challenges for designers of imaging optics. For example, unlike synchrotron X-ray sources, neutron sources are relatively large, of the order of 10 mm, diffuse, and weak. Given that the refractive index of neutrons in most materials is very close to one, preserving and shaping neutron beams is a difficult task.

Neutron focusing optics suitable for imaging applications have been demonstrated based on either refraction [15,16,17] or reflection [18]. Refraction optics are chromatic [19], thus limiting imaging to a particular wavelength, while most neutron imaging techniques can use or even require polychromatic beams. Mirror-based imaging optics are achromatic and are commonly used in X-ray telescopes and microscopes, following Hans Wolter [20]. We previously demonstrated axisymmetric neutron imaging mirrors based on similar designs [18,21,22,23].

Despite these developments, the optics that are suitable for polychromatic neutron imaging have relatively short focal lengths, and high solid-angle coverage has not yet been implemented, partially due to the complexity of the fabrication of high-precision mirrors and their novelty for neutron applications. Therefore, most existing neutron-imaging instruments are based predominately on a pinhole camera geometry, where a collimated neutron beam from a small aperture with a diameter *D* illuminates a sample at a distance *L* and generates an image at the detector placed close to the sample. The spatial resolution of such systems improves with beam collimation (measured as the L/D ratio) and a shorter sample-to-detector distance. However, a larger L/D ratio comes at a high cost of lower neutron flux by restricting the solid angle of the source viewed from the sample. Moreover, the detector needs to be placed very close to the sample to reduce geometric unsharpness. Although improvements of spatial resolution have been reported by optimizing instruments at high-flux sources and using high-efficiency detectors, achieving sub-10 μm resolution requires that the sample-to-detector distance is very small [24,25,26,27,28,29,30,31,32,33,34]. The need for placing samples in close proximity to a detector is very limiting for samples that require, for example, a bulky sample environment (such as the magnetic field, non-ambient temperature, or radiation shielding). Our developments of neutron focusing mirrors could solve this and some other problems related to the basic pinhole camera design of neutron imaging.

Note that pinhole cameras were replaced by microscopes and telescopes hundreds of years ago for visible light and decades ago for electrons and X-rays (including by Wolter mirrors in the case of X-rays [35]). We are striving to achieve the same for neutrons. For example, we have recently demonstrated depolarization imaging with the help of Wolter mirrors [7]. In that experiment, a 1 mm${}^{3}$ sample was placed inside a high-pressure cell, and the ferromagnetic transition was measured with the help of depolarization neutron microscopy with a resolution of 100 μm. Such high spatial resolution was not previously possible for depolarized neutron imaging precisely because a bulky spin analyzer has to be placed between a sample and a detector, resulting in significant blurring of the image and a spatial resolution of 1 mm. Wolter mirrors, however, allow for large sample-to-detector distances without degrading the resolution, as explained below.

**Figure 1:**
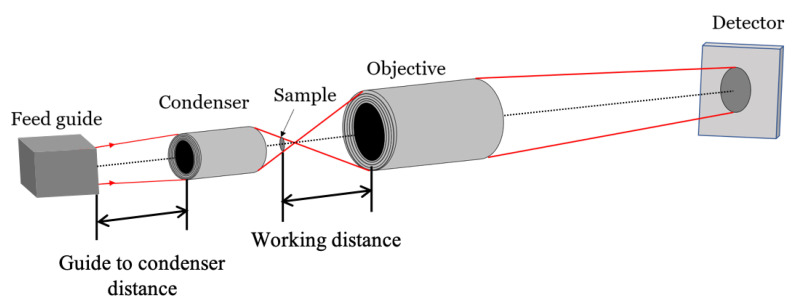
Schematic of a neutron microscope with a condenser and objective mirrors. Red lines trace edge rays through the system, and the dotted line is the optical axis. The sample-to-objective distance is commonly referred to as the working distance in a microscope.

Our previous work was concerned mainly with the image-forming mirrors. Here, we provide example designs of an entire microscope, including the source (feed guide), the condenser, and the image-forming objective, as shown in Figure 1. The importance of the condenser was emphasized in our recent experiments [7,36]. The system design of all the elements of the microscope is important to optimize its performance. As an example, we use the cold neutron-imaging instrument (CNII) located at the neutron guide NG6 at the NIST Center of Neutron Research (NCNR). We design the neutron microscopes with a magnification of 10 that should achieve a spatial resolution of less than 10 μm. In addition to imaging applications, we find that Wolter type III mirrors are best suited for neutron beam transport, where short mirrors could replace much longer focusing guides. Small-angle scattering and spin-echo neutron instruments could also benefit from our developments, although these applications are not considered here in detail.

**Figure 2:**
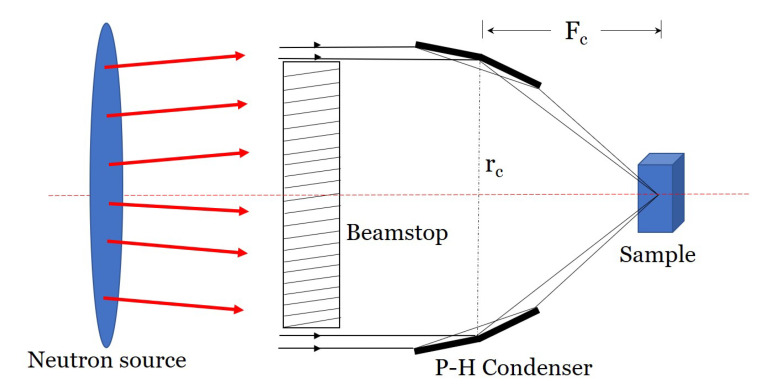
Schematic of the guide condenser system of a neutron microscope. The condenser is placed between the guide and the sample. P-H, paraboloid and hyperboloid.

Specifically, we design and compare objectives based on three types of Wolter mirrors, types I, II, and III, which consist of pairs of confocal, conic-section mirrors that are largely free of spherical, coma, and chromatic aberrations. Incident rays are reflected from the internal surfaces of both mirrors in Wolter I systems (see Figure 2). In Wolter II systems, the rays are reflected from the internal surface of the primary mirror followed by the external surface of the secondary mirror, analogous to the Cassegrain telescope. In Wolter III systems, the first reflection is from the external surface of the primary mirror, and the second reflection is from the internal surface of the secondary mirror [35]. All three systems are described and illustrated below. The performance of all three systems is largely similar concerning the throughput and resolution, although Wolter I is the best design overall. The use of Wolter type I mirrors for neutron imaging has been analyzed in detail and demonstrated, including the analysis of the intensity gain, field of view, and prevalent field-curvature aberrations [18,22]. Subtle differences between the three types of Wolter systems make the detailed comparison of their performances a difficult task.

## 2. Basic Design Considerations and Methods

The optical design of a neutron microscope is shown schematically in Figure 1. Like any microscope, the neutron microscope consists of a condenser optic and an image-forming objective optic. Neutrons are focused at the specimen by the condenser, collecting a diverging neutron beam at the sample. The objective focuses the transmitted beam on the detector. Unlike the pinhole camera system, which requires adaptation of the pinhole to the resolution needs of the study, the Wolter mirror microscope operates with a much more divergent neutron beam. The divergence increases the flux illuminating the sample by using a larger solid angle portion of the source than that of high L/D settings. The divergence ensures that the beam passing through the sample is subsequently reflected by the objective mirrors. A tightly collimated beam would miss the mirrors.

Incident neutrons are reflected from mirrors only at the grazing-incidence angles smaller than the material- and wavelength-dependent critical angle. The critical angle can be increased by coating the surface with multiple layers, generally referred to as neutron supermirrors. The supermirror parameter, the *m*-number, is the ratio of the critical angle of the multilayer to that of Ni. Thus, *m* = 1 corresponds to a natural Ni surface. For example, ${}^{58}$Ni has a critical angle equivalent to *m* = 1.2. The critical angle is proportional to the wavelength and the *m*-number.

To build a high-throughput microscope, the condenser and the objective should be designed simultaneously. This is because the condenser determines the size and the divergence of the beam, which is later intersected by the objective. In other words, the numerical apertures (NA) of the two optics have to match (the NA is the ratio of the mirror diameter to the focal length). Another important factor is the sample-to-objective distance, or the working distance (see Figure 1). The closer the sample to the mirror, the better resolution can be achieved. The reason is that the imperfections of the mirrors, called figure errors, create a distribution of reflected rays with a certain angular spread. The angular spread results in a sharper point-spread function at shorter working distances. However, the minimum working distance (the sample-to-objective distance) is set by both the desire to insert a sample environment around the sample and the critical angle of the objective, which sets the divergence of the beam passing through the sample field of view.

We designed the microscopes suitable for cold neutron-imaging beamlines. As an example, we used the parameters of the NG6 guide at NIST after it is upgraded with *m* = 2 supermirror neutron guides. Neutrons are moderated using a liquid deuterium moderator at 20 K, and the neutron spectrum is approximated by a Maxwell–Boltzmann distribution with a characteristic temperature of 40 K, corresponding to a peak wavelength of 5 Å. We used the 3 Å to 10 Å wavelength band, for which the beam divergence at the end of the guide will have less than 2° of divergence at the 6 cm × 10 cm guide exit. These parameters are in general similar to many existing cold neutron-imaging facilities. Although the performance will depend on these initial parameters, the overall methods and conclusions will be suitable in general for similar beamlines.

As will be clear below, the geometrical optics of Wolter mirrors is rather complex, and the design requires careful ray-tracing simulations. We performed the ray-tracing simulations using McStas [37,38]. We used $10^{6}$ initial rays for the design optimization and $10^{9}$ rays for the resolution calculations. The point of the simulations is to analyze the performance of the whole system (the condenser plus the objective) in terms of the throughput and the resolution. The throughput is analyzed by calculating the relative intensity, which is the ratio of the neutron currents (neutrons per second) at the image plane to that at the end of the neutron guide [39]. In ray-tracing, the intensity is determined as the weighted ratio of the number of rays at the detector to that at the source [37,38]. The resolution is determined with the help of the modulation transfer function (MTF). We modeled by ray-tracing an image of a test sample with sharp edges, from which the edge-spread function and the MTF were calculated.

## 3. Design of Wolter Microscopes

All microscopes designed here have Wolter type I condensers, which consist of axisymmetric confocal paraboloid and hyperboloid (P-H) mirrors. The focal point of the paraboloid is confocal with that of the hyperboloid. The sample is positioned at the second focal point of the hyperboloid [20,40].

The geometry of the condenser optics determines the phase space (i.e., the size and divergence) of the beam illuminating the sample. The geometry of the condenser itself is determined by three parameters: mirror diameter, mirror focal length, and total mirror length. The focal length ($F_{c}$) of the condenser is the distance between the sample position and the P-H mirror intersection. The focal length is related to the grazing incidence angle ($\theta_{i}$) at the intersection, $\theta_{i}=1/4\tan^{-1}(\frac{r_{c}}{F_{c}})$, where $r_{c}$ is the P-H mirror intersection radius. $\theta_{i}$ strongly influences the collection efficiency of the mirrors. Smaller $\theta_{i}$ reduces the collecting area, eventually reducing the intensity at the sample position. A longer focal length also decreases $\theta_{i}$, as well as the beam divergence. The guide-to-condenser distance also influences neutron intensity at the image plane. Therefore, to design the optimized microscope, it is important to design an efficient condenser. The following constraints are used in this paper:

1. The maximum sample diameter is 10 mm.
2. The minimum inner diameter of the condenser is greater than or equal to 20 mm.
3. The minimum inner diameter of the objective is greater than 20 mm.
4. All mirrors of the condenser must have the same axial length of 300 mm.
5. The thickness of each mirror is 1 mm.
6. The magnification is 10.

For the ease of optimization and comparison, no nesting was used in any of the figures below. We only present the results of the calculations of single mirror shells. Once the best design is chosen, it is straightforward to introduce additional coaxial nested mirrors [21].

### 3.1. Wolter I Microscope

**Figure 3:**
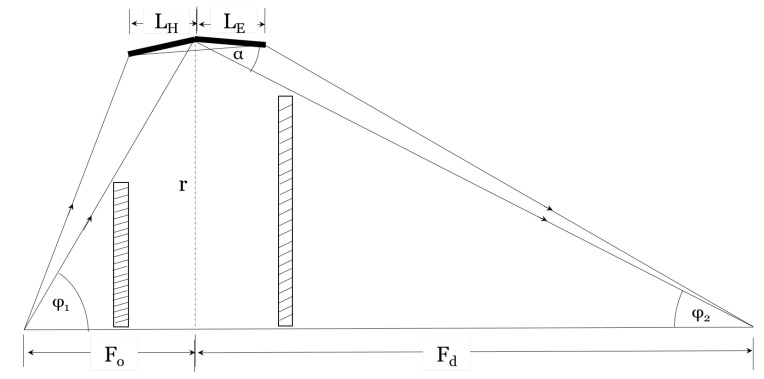
The geometry of the Wolter I objective. Rays from the optical axis are shown for demonstration.

The Wolter type I microscope consists of a Wolter type I condenser with a P-H mirror pair and a Wolter type I objective with a hyperboloid-ellipsoid (H-E) mirror pair. For simplicity, the schematics of the condenser and the objective are shown separately in Figure 2 and Figure 3, respectively. The magnification is defined as the ratio of the distances between the detector and the objective ($F_{d}$) and the objective and the sample ($F_{o}$), also referred to as the objective focal length in this paper, $M=F_{d}/F_{o}$. The condenser and the objective are confocal at the sample position.

**Figure 4:**
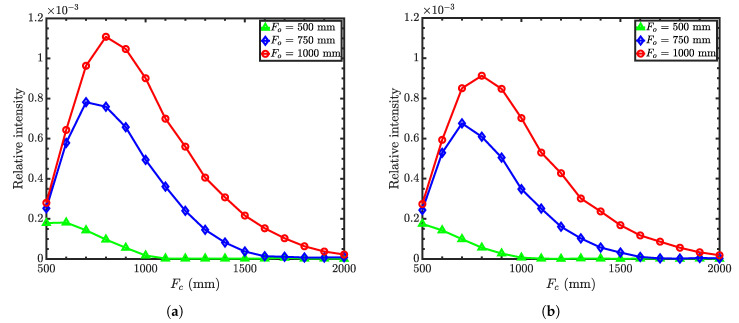
Intensity at the detector (relative to the source) for different objective focal lengths of Wolter I microscopes. Objective focal lengths *F*_0_ = 500 mm (triangles), 750 mm (diamonds), and 1000 mm (circles). (**a**) Guide-to-condenser distance = 500 mm. (**b**) Guide-to-condenser distance = 1000 mm. The simulations were conducted with 10^6^ rays.

**Figure 5:**
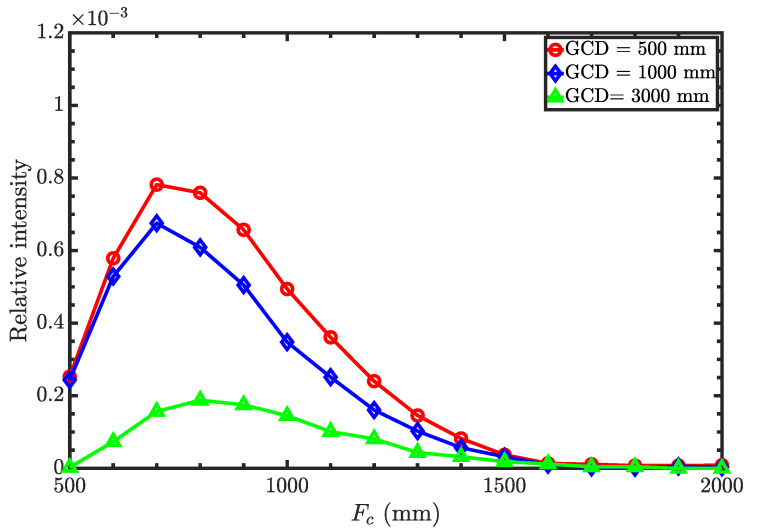
Relative intensity as a function of condenser focal length for three different guide-to-condenser distances (GCD) (Wolter I objective). $F_{0}$ = 750 mm.

As mentioned earlier, both the condenser and the objective are optimized simultaneously to achieve the optimized microscope parameters. Figure 4a,b shows the relative neutron intensity as a function of condenser focal length for different objective focal lengths, with the guide-to-condenser distance (GCD) of 500 mm and 1000 mm, respectively. Relative intensity refers to the ratio of the intensity at the image plane to that of the source. It is clear that the relative intensity increases with increasing objective focal length. However, the spatial resolution of the microscope decreases with increasing focal length because of the geometrical imperfections of the surface profile or figure errors. Hence, the distance between the sample and the objective should be as short as possible. As a compromise, we chose the objective focal length to be 750 mm. Then, the intensity is optimized for GCD as a function of condenser focal length for an objective focal length of 750 mm. Figure 5 shows the relative intensity as a function of the condenser focal length for the guide-to-condenser distances of 500 mm, 1000 mm, and 3000 mm with an objective focal length of 750 mm. Here, we obtain the maximum intensity with a GCD of 500 mm at the condenser focal length of around 700 mm. Note that Figure 4 and Figure 5 show the results without any nesting.

**Figure 6:**
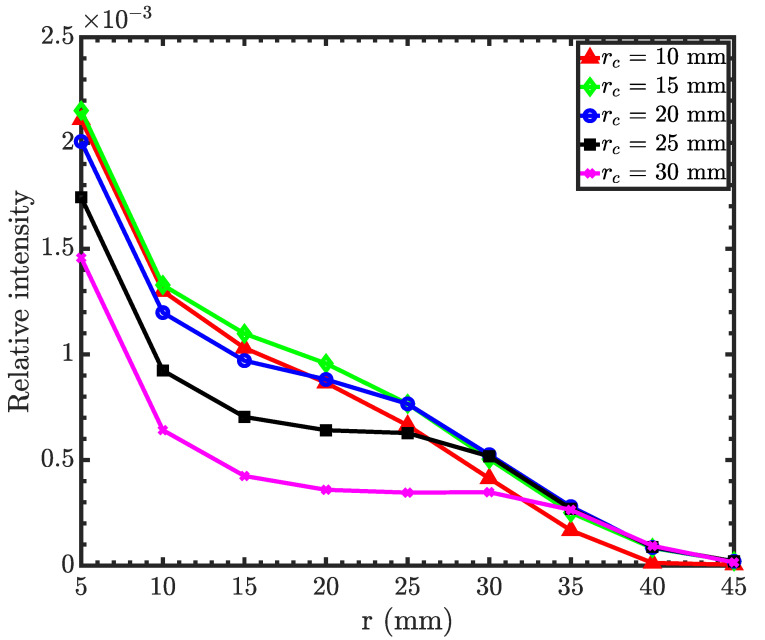
Relative intensity as a function of the objective mirror minimum radius for different condenser minimum radii. The condenser and objective focal lengths are 750 mm (Wolter I objective).

To maximize the collection efficiency, we simultaneously optimized both radii of the condenser and the objective. Figure 6 shows the relative intensities at the image plane as a function of an objective mirror radius for different condenser mirror radii. As seen from the figure, smaller radii for both the condenser and the objective provide maximum neutron intensity. Neutrons will not reflect from mirrors that are greater than 40 mm in radius because the grazing incidence angle will be larger than the critical angle. Furthermore, the minimum mirror radius is limited to 10 mm due to fabrication constraints. The condenser can accommodate up to eight nested P-H mirror pairs with focal lengths of 750 mm, and the objective up to five nested H-E pairs with the same focal length. Other design parameters are a condenser minimum radius of 10 mm, an objective minimum radius of 15 mm, a condenser maximum radius of 35 mm, and an objective maximum radius of 35 mm. See Table A1 for the complete list of design parameters.

### 3.2. Wolter II Microscope

**Figure 7:**
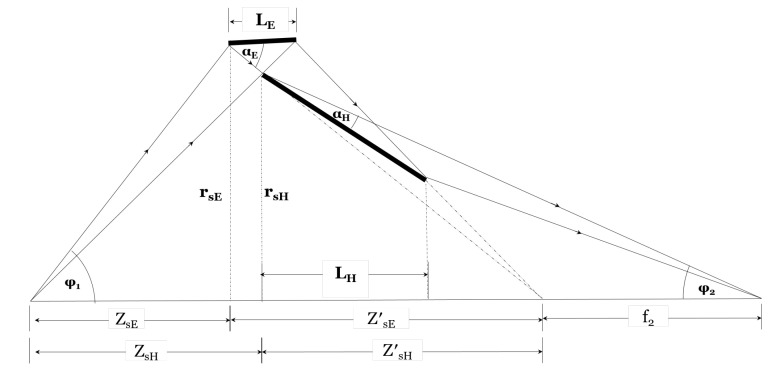
Geometry of Wolter II optics. Rays from the optical axis are shown for demonstration.

The Wolter type II microscope consists of the same condenser as the Wolter I microscope (shown in Figure 2) and a Wolter type II objective (shown in Figure 7) with a pair of ellipsoid-hyperboloid mirrors. Incident neutrons reflect from the inner surface of the ellipsoid and, then, the outer surface of the hyperboloid. The double-reflected neutrons reach the second focus of the hyperboloid where the detector is positioned. The magnification $M=\phi_{1}/\phi_{2}$, where $\phi_{1}$ is the incident-ray angle at the sample position and $\phi_{2}$ is the reflected-ray angle at the detector along the optical axis. From the geometry shown in Figure 7, the grazing incidence angle at the front-edge of the hyperboloid ($\alpha_{H}$) is used to determine the common focus of the E-H mirrors using Equation (1) as:

(1) $$f_{EH}=Z_{sE}+Z_{sE}^{\prime }=Z_{sE}+\frac{Mr_{sE}}{2M\alpha_{H}+\phi_{1}}$$

where $r_{sE}$ is the front-edge radius of the ellipsoid and $Z_{sE}$ is the sample-to-objective distance (the working distance). The grazing incidence angle of the ellipsoid ($\alpha_{E}$) can be expressed in terms of $\phi_{1}$, *M*, and $\alpha_{H}$ as: $\alpha_{E}=\left(\left(M+1\right)/2M\right)\phi_{1}+\alpha_{H}$. Equation (1) shows that for given *M*, $Z_{sE}$, and $\phi_{1}$, the shape of the H-E mirrors depends on $\alpha_{H}$. Since $\alpha_{H}$ and $\alpha_{E}$ are not equal, the multilayer coating of the primary and secondary mirror segments should be different to produce an efficient objective. From the geometry, the front-edge position of the hyperboloid $\left(Z_{sH}\right)$ is determined by Equation (2) as:

(2) $$Z_{sH}=\frac{f_{EH}r_{sE}}{r_{sE}+\left(f_{EH}-Z_{sE}\right)\phi_{1}^{\prime }}$$

where $\phi_{1}^{\prime }=r_{eE}/Z_{eE}$. $r_{eE}$ and $Z_{eE}$ are the back edge radius of the ellipsoid and the distance between the back edge of the ellipsoid and the sample, respectively. Thus, the front-edge radius of the hyperboloid is $r_{sH}=\phi_{1}^{\prime }Z_{sH}$. The position of the image plane at the downstream focus of the hyperboloid can be found using Equation (3) as:

(3) $$f_{2H}=f_{EH}+f_{2}=f_{EH}+\frac{2M^{2}r_{sH}\alpha_{H}}{\phi_{1}\left(2M\alpha_{H}+\phi_{1}\right)}$$

These type II Wolter mirrors are designed based on the front-edge grazing incidence angle of the hyperboloid, $\alpha_{H}$ = 5 mrad. With smaller $\alpha_{H}$, the flux collection efficiency will be lower due to the narrow annular aperture. At large $\alpha_{H}$, the intensity drops as the reflectivity decreases above the critical angle. Therefore, $\alpha_{H}$ = 5 mrad is chosen, which gives the maximum flux collection.

**Figure 8:**
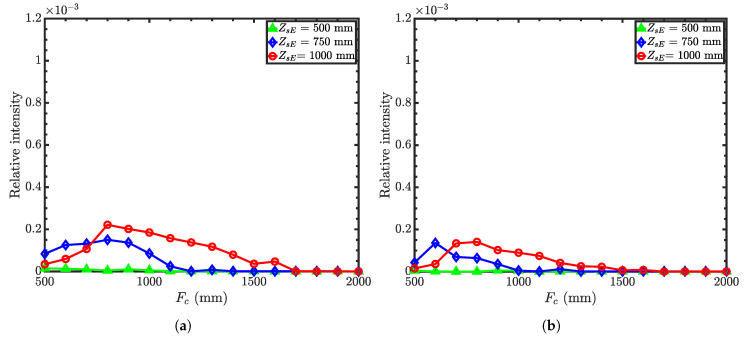
Relative intensities for the Wolter II microscope as a function of condenser focal length with an objective focal length of 500 mm, 750 mm, and 1000 mm. (**a**) Guide-to-condenser distance GCD = 500 mm; (**b**) GCD = 1000 mm. Simulations used 10^6^ rays. Here, the working distance is *Z_sE_*. The vertical scale is the same as in Figure 4 for the ease of comparison.

Figure 8 compares the relative intensities as a function of the condenser focal lengths for different working and guide-to-condenser distances. Similar to the Wolter I microscope, calculations show that neutron intensity is higher for a shorter guide-to-condenser distances. Furthermore, the desired spatial resolution limits the working distance. Hence, suitable Wolter II microscope parameters are as follows: condenser focal length of 750 mm with a working distance of 750 mm and a guide-to-condenser distance of 500 mm.

**Figure 9:**
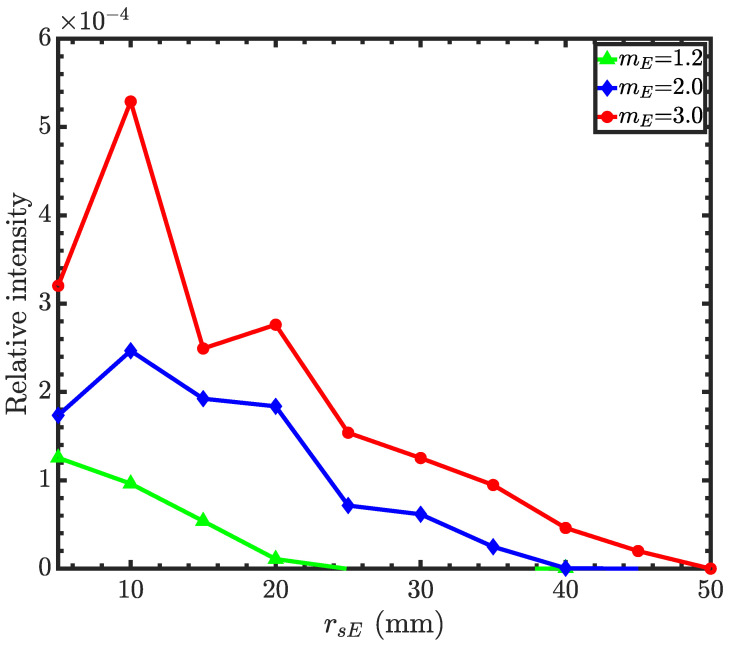
Relative intensities for the Wolter II microscope as a function of the ellipsoid mirror front-edge radius with different ellipsoid mirror coatings. The hyperboloid supermirror is *m*-1.2. The condenser and objective focal lengths are 750 mm. The non-monotonic decay of the intensity with the radius is a result of statistical fluctuations. Simulations were conducted using $10^{6}$ rays.

For Wolter II and III, the collection efficiency depends on the primary mirror coating of the objective (ellipsoid for Wolter II). Hence, we optimized the collection efficiency as a function of the ellipsoid front-edge radius for different mirror coatings. The hyperboloid mirror coating is *m*-1.2 for all cases. Figure 9 shows relative intensities as a function of ellipsoid front-edge radii ($r_{SE}$) with different mirror coatings. The figure shows that the ellipsoid mirror coating of 1.2 results in a smaller objective mirror. This is because the ellipsoid with the $m_{E}$ = 1.2 coating cannot reflect neutrons beyond a 20 mm radius. Such small mirrors are both difficult to fabricate and low in throughput. With the *m*-3 coating, the mirror size is comparable to that of the Wolter I mirror. In Table A1, we report the design parameters of a Wolter type II objective with the ellipsoid *m*-3 supermirror and hyperboloid *m*-1.2 supermirror with five nested objective mirror pairs. We decided to use the same condenser for all Wolter I, II and III microscopes, as reported in Table A1.

### 3.3. Wolter III Microscope

**Figure 10:**
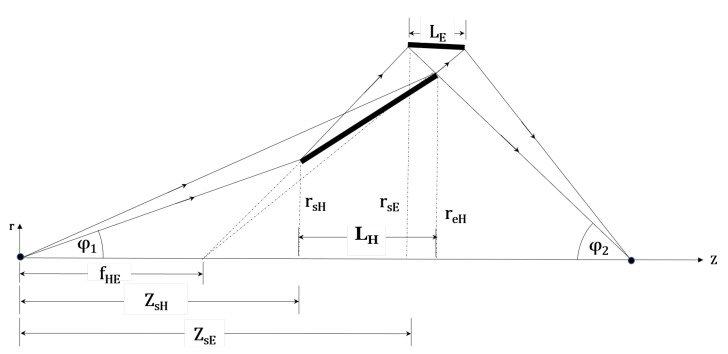
Geometry of the Wolter III optics. Rays from the optical axis are shown for demonstration. $Z_{sH}$ is the working distance.

The Wolter type III microscope consists of the same condenser as before and a Wolter type III objective that consists of a coaxial, confocal hyperboloid and ellipsoid; see Figure 10. Neutrons transmitted by the sample reflect from the outer surface of the hyperboloid and then the inner surface of the ellipsoid. These neutrons then reach the second focus of the ellipsoid where the image is formed. The magnification is defined as the ratio of the incident ray take-off angle ($\phi_{1}$) and the angle of the reflected ray with the optical axis ($\phi_{2}$). The grazing incidence angle at the front-edge of the hyperboloid ($\alpha_{H}$) is used to determine the common focus, as well as the shape of the ellipsoid using Equation (4) as:

(4) $$f_{HE}=Z_{sH}-\frac{r_{sH}}{2\alpha_{H}+\phi_{1}}$$

where $r_{sH}$ is the front-edge mirror radius of the hyperboloid, $Z_{sH}$ is the sample-to-mirror distance or the working distance, and $\phi_{1}$ the incident ray take-off angle with the optical axis. The ellipsoid grazing incidence angle $\alpha_{E}$ is related to the hyperboloid grazing incidence angle $\alpha_{H}$, the magnification *M*, and the incident ray take-off angle $\phi_{1}$: $\alpha_{E}=\left(\left(M+1\right)/2M\right)\phi_{1}+\alpha_{H}$. Using *M*, $Z_{sH}$, and $f_{HE}$, the front-edge position of the ellipsoid is determined by:

(5) $$Z_{sE}=\frac{Z_{sH}+L_{H}+\frac{M}{\phi_{1}}\left[\frac{f_{HE}r_{sH}}{Z_{sH}-f_{HE}}+r_{eH}\right]}{1+\frac{M}{\phi_{1}}\left[\frac{r_{sH}}{Z_{sH}-f_{HE}}\right]}$$

where $r_{eH}$ and $L_{H}$ are the back-edge radius and the length of the hyperboloid, respectively. Equation (5) confirms that the back-edge of the hyperboloid will not block rays reflected by the ellipsoid. The optimal length of the hyperboloid, $L_{H}$, was determined by ray-tracing, which showed that $L_{H}$ significantly affects image quality. We found that the best image quality is achieved for $L_{H}\approx$ 0.4 m. The front-edge radius of the ellipsoid is determined as:

(6) $$r_{se}=\frac{r_{sH}\left(Z_{sE}-f_{HE}\right)}{Z_{sH}-f_{HE}}.$$

The position of the image plane at the downstream focus of the ellipsoid is found by:

(7) $$f_{2E}=Z_{sE}+\frac{Mr_{sE}}{\phi_{1}}$$

The length of the ellipsoid mirror is determined by trial and error. As shown in Figure 10, the geometry dictates the length of the ellipsoid for the ideal case of rays originating at the focal point. However, ray tracing showed that slightly longer mirrors improved collection efficiency on account of off-axis rays.

**Figure 11:**
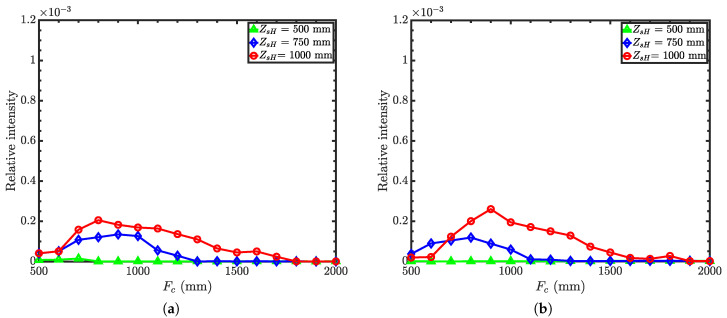
Relative intensity of the Wolter III microscope as a function of condenser focal length with an objective focal length of 500 mm, 750 mm, and 1000 mm. (**a**) Guide-to-condenser distance = 500 mm; (**b**) guide-to-condenser distance = 1000 mm. Here, the working distance is *Z_sH_* The vertical scale is the same as in Figure 4 and Figure 8 for the ease of comparison.

Figure 11 compares relative intensities as a function of condenser focal lengths for different working distances with guide-to-condenser distances of 500 mm and 1000 mm. Similar to Wolter I and II microscopes, the intensity is higher for the shorter guide-to-condenser distance. Compromising again between the desired spatial resolution and intensity, we find optimized parameters for the Wolter III microscope from Figure 11: condenser focal length of 750 mm with a working distance of 750 mm and a guide-to-condenser distance of 500 mm.

**Figure 12:**
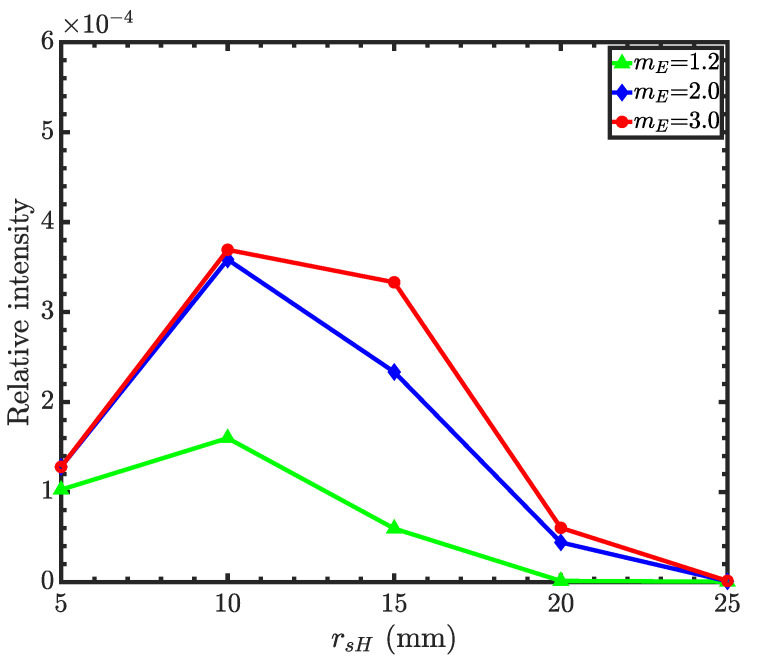
Relative intensity for a Wolter III microscope as a function of hyperboloid mirror front-edge radius with different ellipsoid mirror coatings. The hyperboloid mirror coating is 1.2.

With optimized condenser and objective focal lengths, the objective mirror radii are found for different m-values of the ellipsoidal supermirrors. The hyperboloid supermirror is *m*-1.2. Figure 12 shows the relative intensity as a function of objective mirror radii. The figure shows that even with an *m*-3 ellipsoidal supermirror, Wolter III mirrors cannot reflect neutrons beyond a 30 mm mirror radius, while the difference between the *m*-2 and *m*-3 supermirrors is insignificant in terms of the intensity gain. Thus, the Wolter III objective is much smaller compared to Wolter I and II. Since such small mirrors are difficult to fabricate and coat with multilayers, we conclude that the Wolter III mirrors are not suitable for imaging purposes. Instead, these mirrors are well suited for beam transfer where long focal lengths are needed and the angular resolution is not as important. We imagine that two or three identical Wolter III pairs can be installed to replace a guide system of tens of meters. Beam choppers would be placed in focal spots between the optics.

## 4. Spatial Resolution and Field Curvature Aberrations

Below, we compare the performance of the neutron microscopes and calculate their resolution. Table A1 gathers the design parameters of the mirrors described above.

**Figure 13:**
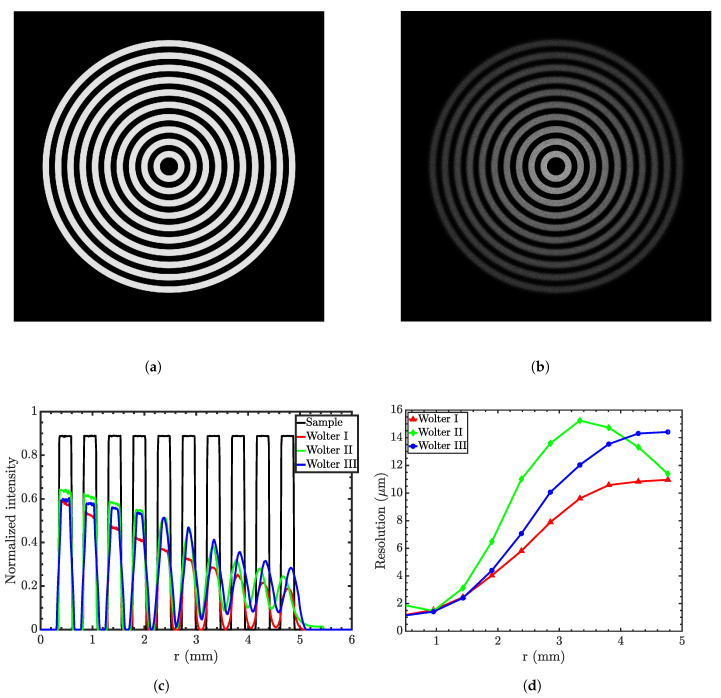
(**a**) Concentric rings sample; (**b**) the magnified image in the Wolter I microscope; (**c**) ideal edge-spread function (ESF) and that obtained by three types of Wolter mirrors; (**d**) the resolution at the focal plane as a function of the distance from the optical axis of the three microscopes. Simulations were conducted using 10^9^ rays.

To test the resolution of the Wolter microscopes, a concentric grating sample (shown in Figure 13a) made of absorbing rings separated by ten transparent rings is simulated. Figure 13b shows a representative magnified image obtained from the Wolter I microscope. Figure 13c shows an edge spread function (ESF) of the ideal concentric rings and those obtained from three types of Wolter mirrors. The edge spread function for each edge is obtained by integrating intensities over the polar angle around the optical axis. The figure shows that at the focal plane, the edges are sharper near the optical axis. As the radius of the rings increases, edges become more spread.

The modulation transfer function (MTF) measures the resolution of an imaging system by representing the signal (i.e., the contrast) as a function of spatial frequency. The MTF is derived from the ESF. The spatial resolution of the system is usually cited as 10% MTF. Figure 13d compares the resolution obtained from three types of microscopes. The figure shows that resolution degrades radially since field curvature dominates the off-axis performance of the microscopes, similar to all other imaging devices based on glancing-incidence optics. The figure also shows that image resolution is in the range of 1 μm at the point of focus.

**Figure 14:**
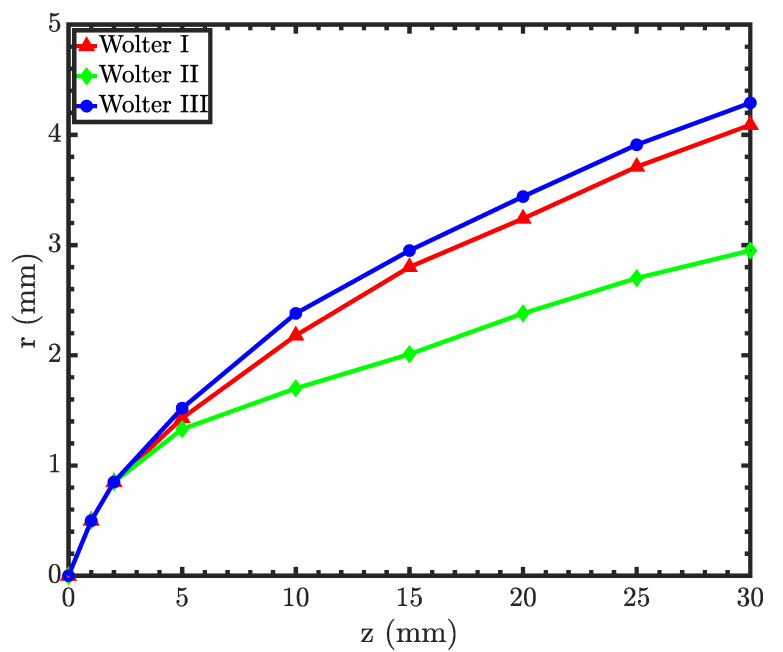
Field curvature of the three optical designs. The r-axis is the radial distance from the optical axis, and the z-axis is the distance from the focal point.

Field curvature aberration can be derived from the MTF measurements. Like other microscopes, Wolter microscopes have Petzval field curvature aberration, which means that the image plane is curved. Figure 14 shows the Petzval field curvature, which is approximately parabolic [22]. Hence, by displacing the detector upstream towards the objective, the resolution of the outer periphery of the sample can be improved, albeit at the sacrifice of resolution at the center. The figure also shows that Wolter I and III have similar field curvatures, while Wolter II has higher field curvature.

## 5. Discussion and Conclusions

The pinhole-based neutron imaging system requires a relatively small source size, while the sample is placed at a very large distance from the source, but very close to the detector. In other words, the spatial resolution of a pinhole system is determined by the L/D ratio, leading to a direct trade-off between the resolution and the signal rate. The magnification in the pinhole-based system is limited due to the proximity between the detector and the sample. These challenges can be addressed by using the proposed neutron microscopes where the source is more divergent than that in the pinhole geometry, resulting in a higher throughput without sacrificing the spatial resolution. Importantly, the resolution of the microscope is determined by the mirrors rather than by the beam collimation, as in conventional pinhole imaging, leading to possible dramatic improvements in the signal rate and resolution. The magnification is obtained by placing the sample very far from the detector, thus leaving space for a bulky sample environment.

In Wolter I microscopes, the lengths of the primary and secondary objective mirrors are assumed to be equal. With a fixed primary mirror length, the length of the secondary mirror depends on the sample-to-detector distance. The difference in length between two mirrors becomes smaller with increasing sample-to-detector distance. Changing the secondary mirror length may improve the off-axis image quality [40]. The grazing incidence angle is another key factor that influences the image quality of the Wolter mirror system. Altering $\theta_{i}$ changes the shape of the mirrors, which influences image quality.

Direct comparison of Figure 4, Figure 8, and Figure 11 shows that the type I microscope offers the best performance in terms of the throughput and resolution. Type II is also inferior in terms of the resolution since it has a stronger field curvature; see Figure 13 and Figure 14. Besides, type II and III optics require higher m coatings and are made of two separate pieces. On the other hand, type I optics can be manufactured as one piece, easing both the manufacturing process and the alignment. This combination of factors makes type I optics the preferred option in general.

In conclusion, we simulated three types of Wolter-type neutron microscopes for high-resolution neutron imaging that will be adapted for future installation at the NIST Center for Neutron Research NG6 beamline and could be adopted at any cold neutron-imaging beamline. We designed a Wolter type I condenser and three types of Wolter objectives suitable for the available space and desired magnification. The condenser is designed to provide optimum flux and phase space for the objective. The objective is designed to provide high-resolution images. All three designs of the objectives were optimized for the same condenser and suitable for a cold neutron spectrum (0.3–1.0 nm). Direct comparison between the three designs shows that the Wolter I geometry is the best optics for imaging.

Let us compare the expected performance of the Wolter type I microscope with that of pinhole imaging systems. High-resolution neutron imaging requires that a geometric unsharpness of about 10 μm can be achieved with 1 cm separation between a detector and a sample and L/D≈ 1000. For the instrument length of 825 cm, the pinhole radius will be 0.4 cm. Assume the neutron flux of $6.8\times 10^{9}$ n/cm${}^{2}$/s at the end of an *m*-2 guide (a reasonable number for high-flux neutron research facilities); 5 Å neutrons will have a divergence of about one degree. Therefore, the neutron current through the pinhole will be ≈$3.4\times 10^{9}$ n/s and the flux at the detector about ≈$5.4\times 10^{6}$ n/cm${}^{2}$/s. For the case of the optics, no pinhole is necessary; see Figure 1. The relative intensity, which is the ratio of the neutron currents (neutrons per second) at the image plane to that at the source (the end of the neutron guide), is about $10^{-3}$; see Figure 5 and Figure 6. For a field of view (FOV) of 1 cm in diameter (see Figure 13) and a source of 6 × 10 cm${}^{2}$, the flux at the detector will be 5.2 × 10${}^{8}$ n/cm${}^{2}$/s (= 6.8 × 10${}^{9}$× 6 × 10/0.5${}^{2}/\pi$). Therefore, the optics gives a factor of a gain of 100 in the neutron flux over the 1 cm diameter FOV for high-resolution imaging. It is straightforward to increase the throughput by utilizing the nesting of coaxial confocal mirrors. Several nested mirrors could increase the gain by up to a factor of a few hundred [18,21,23,39].

Thus, Wolter microscopes could produce gains of well over two orders of magnitude in time resolution over the conventional pinhole optics. At the same time, Wolter mirrors allow the use of bulky sample environmental equipment and additional neutron optical components such as neutron spin analyzers, while maintaining high spatial resolution. This approach and the conclusions are generally applicable for any neutron imaging beamline. Hence, such microscopes could become a game changer and essential tools at high-resolution neutron imaging facilities.

## Appendix A.

**Table A1:** Design Parameters for Wolter Type I, II, and III Microscopes with 10× Magnification.

Parameter	Condenser	Wolter I	Wolter II	Wolter III
Primary mirror outer shell diameter (mm)				
Front	64.34	55.62	60.00	60.00
End	60.00	60.00	61.55	66.99
Secondary mirror outer shell diameter (mm)
Front	60.00	60.00	58.72	67.06
End	46.28	61.02	57.12	70.64
Primary mirror inner shell diameter (mm)				
Front	29.31	38.154	53.68	-
End	27.32	39.14	55.16	-
Secondary mirror inner shell diameter (mm)
Front	27.32	37.34	53.26	-
End	21.08	36.31	47.14	-
Mirror length (mm)
Primary	300	100	100	400
Secondary	300	100	400	100
Mirror coating (m-number)
Primary	2.0	1.2	3.0	1.2
Secondary	2.0	1.2	1.2	3.0
